# Divergent trends in the incidence and mortality of acute myocardial ischaemic syndrome, especially in women. Evidence from Finland in 1996–2021

**DOI:** 10.1080/07853890.2024.2424455

**Published:** 2024-11-26

**Authors:** Atte Kallström, Ida Holopainen, Oleg Kambur, Markus Perola, Veikko Salomaa, Aki S. Havulinna, Markus Ramste, Juha Sinisalo

**Affiliations:** a Heart and Lung Center, Helsinki University Hospital, University of Helsinki, Helsinki, Finland; b Department of Public Health and Welfare, Finnish Institute for Health and Welfare (THL), Helsinki, Finland; c Clinical and Molecular Metabolism Research Program, Faculty of Medicine, University of Helsinki, Helsinki, Finland; d Institute for Molecular Medicine Finland, FIMM-HiLIFE, Helsinki, Finland

**Keywords:** epidemiology, acute myocardial ischaemic syndrome, incidence, mortality, women

## Abstract

**Objective:**

Although the incidence and case fatality (CF) of acute myocardial ischaemic syndrome (AMIS) have declined in recent decades, some studies have suggested a potential stagnation in this decline. We examined if a similar development in AMIS trends can be observed in Finland from 1996 to 2021 among persons aged 35–74 years.

**Methods:**

We linked Finnish country-wide Hospital Discharge- and Causes of Death- Registers covering the first non-fatal and fatal myocardial ischaemic events (total 69 906 442 person-years at risk). We analyzed the incidence, mortality, and 28-day CF and their trends using negative binomial, Poisson, segmented and logistic regression adjusting for age and sex.

**Results:**

The analysis consisted of 186 489 non-fatal and 72 907 fatal myocardial ischaemic events. AMIS incidence declined in men (annual percentage change (APC) −2.0%) and in older women (APC of 55–64 years −1.5%; 65–74 years −3.3%) during the study period. However, the incidence decline slowed down over the last decade in oldest age groups and stopped overall in women. Incidence was unchanged during the study period in younger women aged 35-54 years. AMIS mortality and CF declined (APC of mortality in men −4.4%; in women −5.0%; APC of CF in men −2.7%; in women −3.3%).

**Conclusions:**

AMIS mortality declined in all groups, but the decline in AMIS incidence slowed down and even stopped in women. Incidence was unchanged during the study period in women aged 35-54 years. These results emphasize the need for further efforts in prevention of cardiovascular disease, particularly in young and middle-aged women.

## KEY MESSAGES

- **What is already known on this topic** – *Coronary heart disease treatment and prevention have improved worldwide in recent decades, which has led to a decline in AMIS mortality and case fatality. However, recent studies from several countries suggest that the incidence decline has stagnated or even started to increase, especially in younger age cohorts.*
- **What this study adds** – *This study showed that the incidence decline has stopped in women aged 35-54 years and has slowed down in older age groups. Together with declining mortality, this results in a growing number of patients living with cardiovascular disease, leading to increased healthcare costs.*
- **How this study might affect research, practice, or policy** – *The results of this study underline the importance of intensifying prevention of cardiovascular disease, with a focus especially on young and middle-aged women.*

## Introduction

Cardiovascular disease is one of the world’s leading causes of death and has been extensively researched [1, 2]. Established modifiable risk factors are hypertension, dyslipidemia, diabetes mellitus, and smoking. Preventive measures that target these risk factors are effective and have a good cost-benefit ratio [3]. With these measures, acute myocardial ischaemic syndrome (AMIS) mortality rates have declined in recent decades in Western countries. Approximately one-third of the total AMIS mortality decline is due to an increase in survival after myocardial infarction [4]. Compared with earlier decades, patients now have fewer and less severe symptoms, and survival rates are higher. However, there are worrying signs that these positive trends may not be sustainable, as shown by data from the US and Australia [5, 6].

In Finland, the mortality rate of coronary heart disease (CHD) has drastically declined in recent decades. From 1960 to 1970 the mortality rate from AMIS in Finland was among the highests in the world. Most of the reduction was due to improving primary and secondary prevention, with particular focus on classical cardiovascular risk factors [7]. A substantial part of the reduction was also achieved by advances in AMIS treatment, including percutaneous coronary intervention and new preventive medications [8]. However, the northeastern part of Finland has a substantially higher burden of CHD incidence and mortality, partly due to genetic, environmental, and socioeconomic aspects [7, 9]. Previous studies have demonstrated that the short-term (CF) and 1-year prognosis of incident MI have improved in both sexes from the mid-1990s to 2002 in Finland [10].

Data on trends in AMIS incidence and mortality rates and CF in recent years is very sparse globally, also in Finland [7, 11]. As the Finnish electronic health care registries offer reliable and comprehensive nationwide data and a long follow-up period [12], we chose to analyze the trends using these registries and compare our findings with available corresponding data from other Western countries. We focused on AMIS trends in the population aged 35–74 years in Finland from 1996 to 2021. We hypothesized that these trends would be declining as AMIS mortality in the Western world has declined in the past and it was previously projected that age-adjusted myocardial ischaemic event rates would decrease in Finland until 2050 [13].

## Methods

### Study population

We collected data from 1996 to 2021 from Finnish country-wide registers (i.e. hospital discharges) from the Care Register for Health Care and deaths from the National Causes of Death Register with complete coverage of non-fatal and fatal myocardial ischaemic events, including sudden, out-of-hospital cardiac deaths [12]. A unique personal identification number that every permanent resident in Finland receives at birth or upon immigration links the data between the registries. Altogether, the analysis consisted of 186 489 non-fatal and 72 907 fatal myocardial ischaemic events.

We defined the annual population as the population on the last day of each year, available from the Finnish Population Information System. At the end of 2021, the population of Finland aged 35–74 years was 2.8 million. The study population contributed 69 906 442 person-years at risk.

The information permit number for the use of the Finnish Cardiovascular Disease Register (CVDR) is THL/3624/6.02.00/2023. Permission to access the original data can be applied for through FINDATA. According to Finnish legislation the ethical approval is not needed for register-based studies [14–17]. Therefore, Ethical Committee approval or Informed consent was not required because of the register-based nature of the study. The study complies with the Declaration of Helsinki.

### Definition of AMIS

We chose to use the term acute myocardial ischaemic syndrome (AMIS) that refers to group of events in which blood flow to the heart suddenly decreases due to epicardial obstructive coronary and non-obstructive causes (eTable 1). AMIS replaces the term acute coronary syndrome (ACS), expanding its scope to include all non-obstructive causes of myocardial ischemic events [18].

We included in our data the non-fatal and fatal myocardial ischaemic events and only the patients’ first event. Non-fatal events consist of those events where the patient did not die within 28 days from the event onset. The event was considered as first if the patient had no previous myocardial ischaemic events recorded in the HDR during the preceding 10 years. If the patient had a second myocardial ischaemic event <28 days from the onset of the previous event, we considered the two events as one and included only the more severe diagnosis code. This is concordant with the definition previously described in the WHO-MONICA project [19].

The total myocardial ischaemic events in our study are non-fatal hospitalizations for MI or unstable angina (ICD-9: 410; ICD-10: I20.0, I21-I22), and fatal events, which include deaths with CHD (ICD-9: 410-414; ICD-10: I20-I25), cardiac arrest (ICD-10: I46), sudden death for unknown reason (Finnish ICD-9: 798, not 7980 A; ICD-10: R96), or unwitnessed death (ICD-10: R98) as the underlying or direct cause of death, or deaths with MI (ICD-9: 410; ICD-10: I21-I22) as the contributing cause of death. We assessed AMIS incidence, mortality, and case fatality for the entire population of Finland by event age, and by event year and sex.

### Age groups

We calculated AMIS incidence, mortality, and CF for the age group 35–74 years. This was further subdivided into smaller age groups (35–44, 45–54, 55–64, and 65–74 years). We examined trends using ten-year age groups because this approach allows us to detect potential differences between age groups more effectively. In CF we combined the two youngest groups into one age group (35–54 years) due to low case counts.

### Statistics

We analyzed incidence and mortality rates observed during the study period using multivariable Poisson or negative binomial (NB) regression models adjusting for *age, sex*, and *year.* We used the NB model instead of the Poisson model if there was evidence of overdispersion, and the NB model had a better fit for the given data. The model selection between Poisson and NB was based on minimizing Akaike information criterion (AIC).

We analyzed case fatality trends using the multivariable logistic regression model, adjusting for *year*, *age*, and *sex*.

We used the segmented regression, with Poisson or NB model, to study potential trend changes in the observed years for AMIS incidence and mortality rate, and CF. We applied the segmentation on the variable *year* with maximum range for breakpoints set from zero to five. In addition, we also adjusted the models for *age*. We selected the Poisson or NB regression model with suitable number of breakpoints by minimizing AIC.

We performed age-standardization of the incidence and mortality rates using the direct method with weights from the 2011–2030 European standard population [20]. This standard population allows further comparison with other publications. The 28-day CF was age standardized using weights derived from the combined age distribution of myocardial infarction and stroke patients in the WHO MONICA project [19]. We used the MONICA weights because the age dis­tribution of myocardial ischaemic events is substantially different and biased towards older age groups compared to the general population. For older age groups we used weights derived previously at the Finnish Institute for Health and Welfare from the MONICA and Morgam projects (eTable 2) [21].

We used R version 4.2.2 for all statistical analyses and considered *p* < 0.05 as statistically significant. All tests were two-tailed. We used package “MASS” (version 7.3-58.1) for negative binomial regression, “segmented” (version 1.6-2) for segmented regression, and “AER” (version 1.2-10) for testing whether there was overdispersion in the Poisson model [22]. The AMIS incidence and mortality rates and CF trends are presented in the figures as 3-year moving averages, the first and the last years are counted as 2-year averages.

The Methods section is also illustrated as a flowchart in the supplement material (eFigure 1). This study has also previously been published as a preprint in medRxiv [23].

## Results

### Overall incidence and mortality

**Figure 1.**
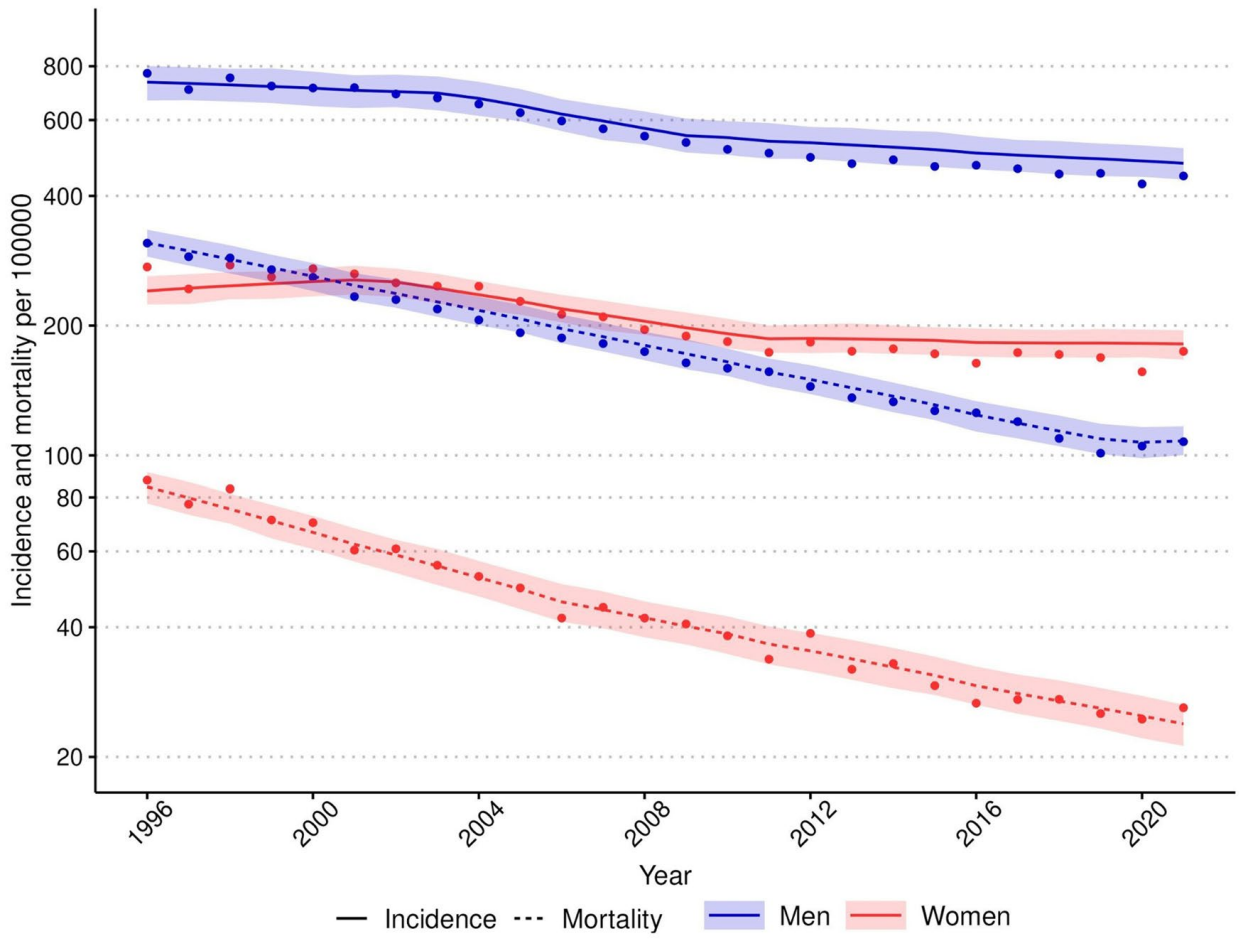
Trends in incidence and mortality rates of acute myocardial ischaemic syndrome in men and women aged 35–75 years, 1996-2021. The rates include the first non-fatal I20.0, I21, and I22 cases and fatal I20-25, I46, R96 and, R98 cases. Age-standardized rates per 100 000 inhabitants were calculated with the 2011-2030 European standard population as the reference. The observed incidence and mortality rates are presented as dots, the segmented (negative binomial) regression model’s predicted values as lines and the segmented regression model’s 95% confidence intervals for predictions as ribbons. The rates are presented on a logarithmic scale.

AMIS incidence and mortality in men and women declined overall during the study period (Figure 1). The decline in mortality was markedly steeper than the decline in incidence. The age-standardized AMIS mortality rate declined by more than two-thirds (in men 65.3% and in women 71.8%) and the incidence rate declined approximately by one-third in men (35.1%) and by one-quarter in women (24.6%).

**Figure 2.**
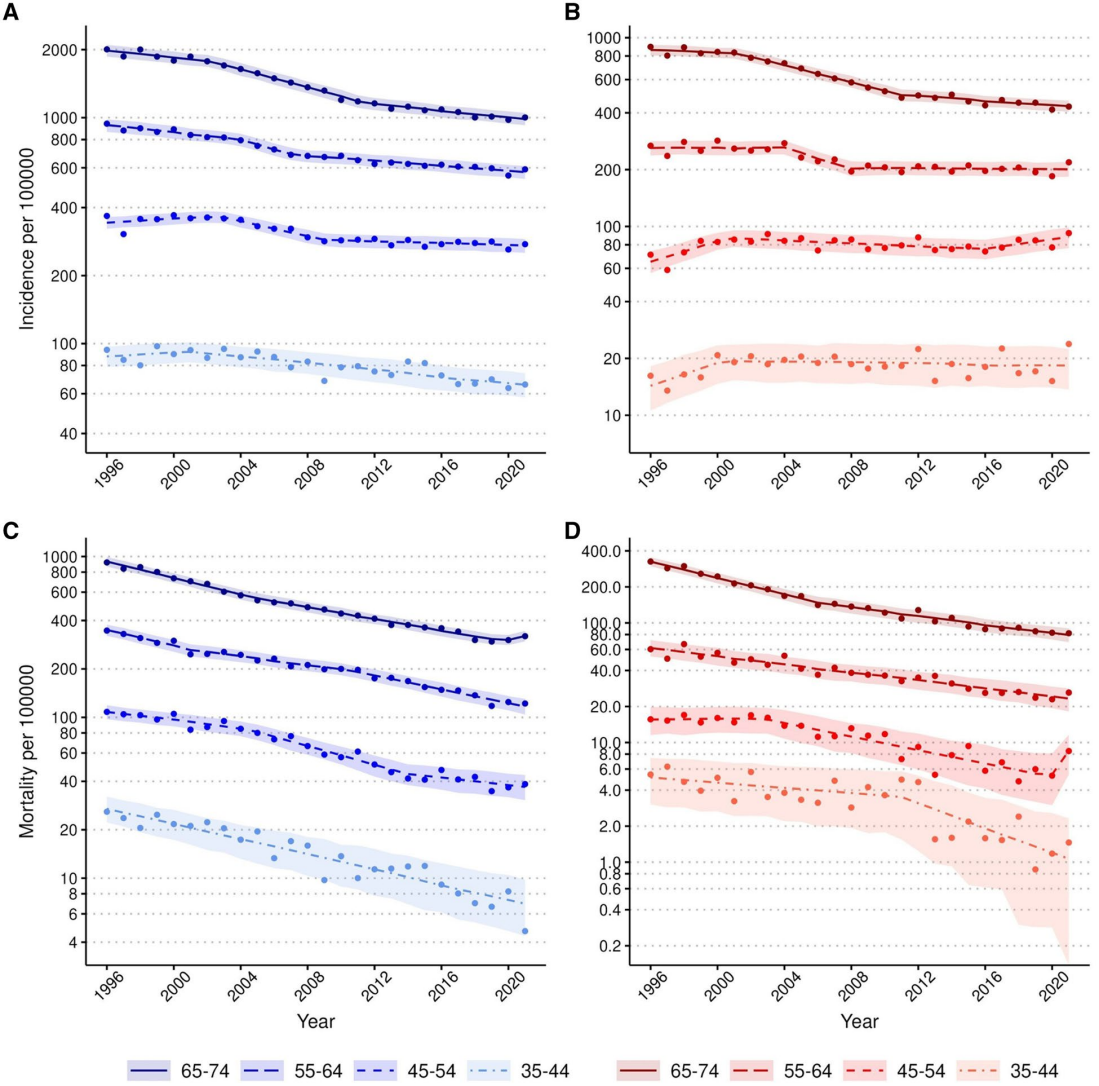
Trends in incidence and mortality rates of acute myocardial ischaemic syndromein men and women by age group, 1996–2021. A) Incidence rates of men. B) Incidence rates of women. C) Mortality rates of men. D) Mortality rates of women. The incidence rates include the first non-fatal I20.0, I21, and I22 and fatal I20-25, I46, R96, and R98 cases. The mortality rates include the fatal cases as mentioned above. Age-standardized rates per 100 000 inhabitants were calculated with the 2011-2030 European standard population as the reference. The observed incidence and mortality rates are presented as dots, the segmented, Poisson, or negative binomial regression model’s predicted values as a line, and the regression model’s 95%-confidence intervals for predictions as ribbons. The rates are presented on a logarithmic scale.

Men had approximately two-fold higher incidence than women in the oldest age group and four-fold higher incidence in younger age groups (35–54 years (Figure 2A and B). Men also had an approximately three-fold higher mortality in the oldest age group (65–74 years and a five-fold higher mortality rate in the other age groups (Figure 2C and D). Annual incidence and mortality changes per age group, number of cases in the age groups, mean age of each group, and p-values of trends are shown in Table 1.

**Table 1.** Average annual changes in acute myocardial ischaemic syndrome incidence and mortality rates, and case fatality from 1996 to 2021. The incidence and mortality rate trends were analyzed using the Poisson or the negative binomial regression model. Case fatality trends were analyzed with a logistic regression model. Rate changes are presented as average annual change percentages during the study period. P-values represent the significance of the trend by study year.

**Incidence**					
Age group (years)	Mean age (years)	Cases (N)	Average annual change (%)	95% CI	P-value
**All**					
35–74*	62.7	259 396	−1.82	−1.96 to −1.69	<1e-16
**Men**					
All (35–74)*	62.0	185 309	−2.01	−2.20 to −1.82	<1e-16
65–74*	69.6	84 763	−3.12	−3.29 to −2.96	<1e-16
55–64*	59.9	61 912	−2.06	−2.23 to −1.89	<1e-16
45–54*	50.4	30 985	−1.40	−1.57 to −1.22	<1e-16
35–44	40.9	7 649	−1.33	−1.63 to −1.04	<1e-16
**Women**					
All (35–74)*	64.7	74 087	−1.66	−1.83 to −1.48	<1e-16
65–74*	70.1	44 294	−3.28	−3.40 to −3.16	<1e-16
55–64*	60.2	20 304	−1.46	−1.70 to −1.21	<1e-16
45–54	50.5	7 818	0.20	−0.10 to 0.50	0.19
35–44	41.0	1 671	0.38	−0.25 to 1.02	0.24
**Mortality**					
**All**					
35–74*	64.2	72 907	−4.57	−4.72 to −4.43	<1e-16
**Men**					
All (35–74)*	64.5	56 900	−4.42	−4.59 to −4.24	<1e-16
65–74*	69.6	30 602	−4.49	−4.68 to −4.30	<1e-16
55–64*	60.4	18 028	−4.05	−4.29 to −3.82	<1e-16
45–54	50.8	6 835	−4.77	−5.09 to −4.45	<1e-16
35–44	40.7	1 435	−5.29	−5.98 to −4.60	<1e-16
**Women**					
All (35–74)*	66.2	16 007	−5.00	−5.21 to −4.72	<1e-16
65–74*	70.1	11 104	−5.50	−5.78 to −5.21	<1e-16
55–64	60.6	3 488	−3.84	−4.28 to −3.39	<1e-16
45–54	50.7	1 104	−4.47	−5.27 to −3.67	<1e-16
35–44	40.9	311	−4.87	−6.36 to −3.40	3.06e-10
**Case fatality**					
**All**					
35–74	62.7	259 396	−2.86	−2.98 to −2.75	<1e-16
**Men**					
All (35–74)	62.0	185 309	−2.71	−2.84 to −2.57	<1e-16
65–74	69.6	84 763	−2.19	−2.37 to −2.01	<1e-16
55–64	59.9	61 912	−2.75	−3.00 to −2.52	<1e-16
35–54	48.5	38 634	−4.45	−4.78 to −4.12	<1e-16
**Women**					
All (35–74)	64.7	74 087	−3.35	−3.58 to −3.12	<1e-16
65–74	70.1	44 294	−3.15	−3.43 to −2.88	<1e-16
55–64	60.2	20 304	−2.79	−3.28 to −2.30	<1e-16
35–54	48.9	9 489	−5.74	−6.51 to −5.00	<1e-16

*Negative binomial regression model was used instead of the Poisson model.

### Incidence trends

The trends in AMIS incidence are presented in Figure 2. In men, the incidence of AMIS declined significantly in all age groups during the study period (APC in men −2.0%, 95% CI −2.2 to −1.8) (Table 1). However, the segmented regression revealed that the incidence decline has slowed down significantly overall in men and in men’s age groups 55-64 and 65-74 years and stopped in age group 45-54 years from around 2007-2012 onwards (eTable 3, eFigure 2 and 3A).

In women, the incidence rate declined significantly overall and in the older age groups (overall APC −1.7, 95% CI −1.8 to −1.5; 65-74 years −3.3%, 95% CI −3.4 to −3.2; 55-64 years −1.5%, 95% CI −1.7 to −1.2). The overall incidence in women did not decline after 2010 and the age-specific incidence rates slowed down in the older age groups from 2008 to 2011 (eTable 3, eFigures 2 and 3B). Importantly, in women aged 35-54 years, AMIS incidence remained the same for over two decades (APC of 35-44 years 0.4%, 95% CI −0.3 to 1.0; 45-54 years 0.2%, 95% CI −0.1 to 0.5). The stagnated or slowed incidence decline is mostly associated with inferior development of non-fatal myocardial ischaemic events compared with the incidence of all myocardial ischaemic events (eTable 4, eFigure 4A–C). The incidence of non-fatal myocardial ischaemic events among young women aged 35–54 years increased during the study period.

Men have higher risk than women to have a myocardial ischaemic event, and increasing age increases the risk to have a fatal event. Increasing age is a stronger risk factor for AMIS incidence in women than in men (eTable 5).

### Mortality trends

The trends in AMIS mortality are presented in Figure 2 and the breakpoints in eFigures 2 and 3C–D. AMIS mortality declined in both sexes and all age groups during the study period (APC of mortality in men −4.4%, 95% CI −4.6 to −4.2; in women −5.0%, 95% CI −5.2 to −4.7). Overall, the annual mortality change was very similar in all age groups. In women, mortality declined most in the oldest age group (APC −5.5%, 95% CI −5.8 to −5.2) while in men, mortality declined most in the youngest age group (APC −5.3%, 95% CI −6.0 to −4.6). However, in younger age groups, especially in women, case numbers are low (Table 1).

Men have higher risk than women to have a fatal myocardial ischaemic event, and increasing age increases the risk to have a fatal myocardial ischaemic event. As in incidence, increasing age is a stronger risk factor for AMIS mortality in women than in men (eTable 5).

### 28-day case-fatality trends

**Figure 3.**
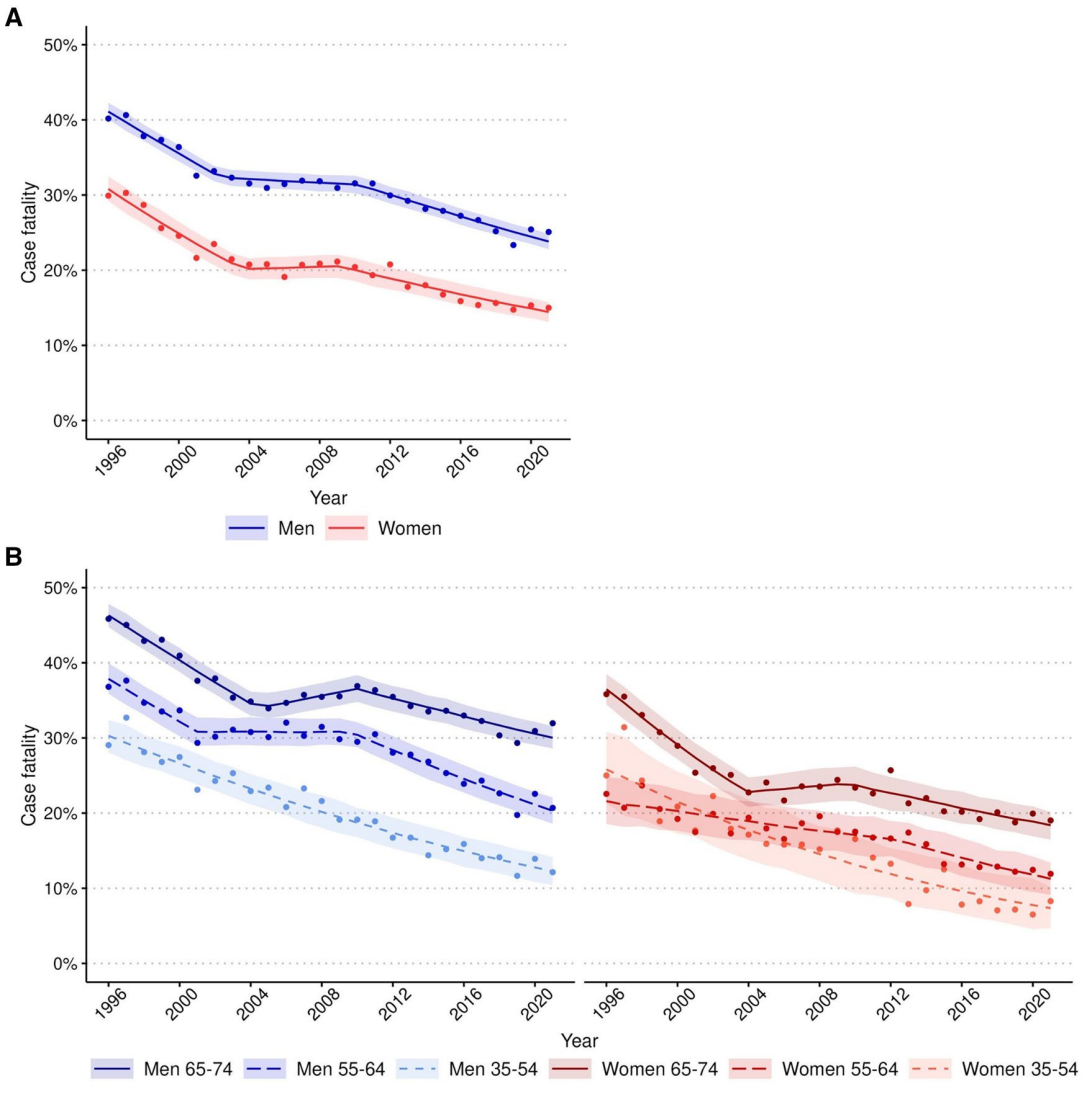
Trends in case fatality of acute myocardial ischaemic syndrome (A) among men and women aged 35–74, and (B) by 10-year age groups, 1996-2021. The case fatality includes the first non-fatal I20.0, I21, and I22 and fatal I20-25, I46, R96, and R98 cases. The case fatality was age-standardized using weights based on the age distribution of observed coronary events in populations participating in the WHO-MONICA project. The observed case-fatality is presented as dots, the segmented or logistic regression model’s predicted values as a line and the regression model’s 95%-confidence intervals for predictions as ribbons.

AMIS CF declined over the study period in men and women (APC of CF in men −2.7%, 95% CI −2.8 to −2.6; in women −3.3%, 95% CI −3.6 to −3.1) (Figure 3A). Women had lower AMIS CF across equivalent age groups than men (Figure 3B). The decline was not linear, particularly in the older age groups. CF stayed the same or even increased a little from around 2001 to 2010, depending on age group and sex (eTable 3 and eFigure 5A and B). The annual decline in CF was most significant in the youngest age group. Men have higher case fatality than women and increasing age increases risk for higher case fatality. Increasing age increases CF equally in both sexes (eTable 5).

## Discussion

This nationwide study of almost 260 000 events revealed a declining incidence of AMIS in the population aged 35–74 years during the study period of 25 years. However, the incidence did not decline in young women aged 35–54 years. Also, in the oldest age groups, the declining incidence trends slowed down during the study period. Furthermore, the overall incidence decline in women stopped in the last decade.

Our study also showed that the mortality rate declined in men and women and, unlike incidence, in all age groups throughout the study period. The rate of decline was about similar in all age groups. The decline in mortality is consistent with an earlier prediction [13]. It is also worth noting that the decline in mortality was much faster than in incidence. CF declined similarly as mortality in both men and women and in all age groups during the study period. The decline in CF was most significant in the youngest age group (35–54 years). The CF was much lower overall in women than in men.

To understand why the incidence of AMIS is no longer steadily declining, we examined the results of the National FinHealth 2017 survey that has gathered information on the health of the Finnish adult population and on the risk factors influencing their health [24]. The FinHealth study showed that the proportion of smokers has declined. The study also showed that the prevalence of elevated LDL has declined, but no changes in low HDL levels were seen. Alcohol abstinence has also increased. However, dietary habits have not improved, obesity as well as the waist-to-hip ratio have increased, and hypertension is still very common in Finland. In summary, except for obesity and diabetes, other AMIS-related risk factors have improved. Also, a more recent study made in 2023 has pointed out that abdominal obesity is still increasing in Finland, especially in working-age women [25].

A factor that could specifically explain the levelling off of the incidence decline in women aged 35-54 years compared to the declining trend of the same aged men may be that diabetes is a stronger risk factor in women than in men [26], considering that diabetes prevalence has also increased [24, 27]. Even as smoking has lost popularity, tobacco exposure, both current and accumulated, also predisposes female smokers more to premature myocardial infarction than men [28]. In addition to these somatic factors, psychosocial factors may play a role. Women under 50 years of age who develop a myocardial infarction have more psychosocial risk factors than same-aged men and depressive symptoms are associated with chronic heart disease events [29].

Studies have shown that primary prevention is implemented better in women than in men, but women receive less invasive measures and less guideline recommended secondary preventive medications than men after myocardial infarction. Although women have a lower risk for recurrent myocardial infarction and a lower rate of 30-day cardiovascular deaths compared to men, women are reported to have a higher rate of long-term all-cause mortality [30].

Other reports have shown that the decline in AMIS trends has slowed down, especially among younger age groups. In France, the hospitalizations for ACS have increased in women aged less than 65 years from 2004 to 2014 while hospitalizations among men have decreased. Factors behind the increase in women’s hospitalization in the study are suggested to be increased obesity and smoking [31]. In an Australian study, the mortality for CHD has levelled off already from the early 1990s to 2006 in population aged 25–54 years. Also, in this study the finding is linked with increased prevalence of traditional risk factors such as diabetes [6].

In the older age groups, the slowing down of the incidence decline in the last decade may be due to exhaustion of optimizing preventive treatment and therapeutic inertia, especially regarding the use of new expensive treatment modalities (e.g. PCSK9, siRNA drugs) that will be utilized in younger age groups [32]. In addition, for all age groups, improving AMIS prevention and reducing mortality beyond optimal treatment of classical risk factors may require a novel avenue of targeting the residual risk [33] that may not be related to classical risk factors but mediated through inflammation and other poorly understood pathomechanisms within the vascular wall [34]. Recently new tools, such as polygenic risk scores, have been developed to predict the residual and genetic risk of AMIS [35].

A factor that may have an overall influence on the development of incidence and CF in the twenty first century is the introduction of sensitive troponins to clinical practice which has made detection of smaller and less dangerous myocardial infarction easier than before. The use of sensitive troponin can also lead to false myocardial infarction diagnoses as troponin concentration can be elevated also for other reasons than myocardial infarction [36]. Additionally, the COVID-19 pandemic may have influenced the incidence and mortality figures of the last two years as COVID-19 is a risk factor for AMIS [37].

Our study showed that AMIS mortality declined steadily in women and men in all age groups during the study period. Similar findings have been made in other recent studies. From 2012 to 2020 the mortality from myocardial infarctions has declined in many EU countries, especially in western Europe and the decline has been fast for both under and over 65 year old population [38]. Also, in Germany ACS incidence has declined in the whole population from 2005 to 2015 and at a same timeframe the in-hospital mor­tality for myocardial infarctions has declined which indirectly indicates that ACS related mortality has declined [39]. Awareness of coronary heart disease may influence earlier seeking of care, which results in lower mortality.

Because AMIS mortality has declined fast in Finland, Finland is included in the moderate-risk region in Europe based on the death risk due to cardiovascular disease, which leaves room for improvement as many countries in Western Europe belong to the low-risk region [40]. Taking this into account, a decline in mortality and incidence could be continuously achieved and improved, especially if treatment methods evolve and if there is more focus on primary and secondary prevention.

We also found out that increasing age is a stronger risk factor for AMIS incidence and mortality in women than in men. This could be explained by the fact that the AMIS incidence and mortality rates are already higher in men than in women at the age of 35 which can be partly explained by protection of estrogen among premenopausal women [41].

Our results showed that CF declined rapidly in men and women, especially in the youngest age group. This positive trend may be explained by the same factors as the decrease in mortality. This can also be influenced by the introduction of sensitive troponins [36]. In addition, we identified a plateau phase in the overall declining CF trend from 2002 to 2010. An explanation for this plateau phase in CF is not known.

Our study was based on comprehensive nationwide Finnish Cardiovascular Disease Register data which covers all symptomatic acute myocardial infarction syndrome incidents in Finland with a long study period of 25 years. However, the study should be interpreted considering certain limitations: As the Finnish health care registers do not provide individual-level clinical information, such as ECGs or troponins, the diagnoses of the cases could not be further verified. This may be a factor that increases the inaccuracy of the dataset. Also, we had no data on cardiovascular risk factors (such as tobacco abuse) that could be incorporated in these analyses so the possible contributing factors behind the development of AMIS trends are hypothetical. Additionally, silent myocardial infarctions are not recorded in the registers, which probably slightly reduces the number of infarctions in the dataset. However, cardiovascular diagnoses in Finnish healthcare registers have been validated and are reliable which increases the confidence in our results [12].

## Conclusions

In the Finnish population aged 35-74 years, the AMIS mortality and CF were steeply declining, whereas the incidence decline is leveling off. Notably, the decline in incidence in older age groups has slowed during the last decade. The overall incidence in women also ceased to decline. Indirectly, this may result in an increase in CHD patients in the Finnish population which increases health care costs. Particularly concerning is the stalled incidence decline in women aged 35-54 years. The findings emphasize the importance of improving primary prevention measures, with a focus especially on young and middle-aged women.

## Supplementary Material

Supplemental Material

## Data Availability

The data that support the findings of this study are available from the corresponding author, M.R., upon reasonable request.
