# Supplementary material for: Divergent trends in the incidence and mortality of acute myocardial ischaemic syndrome, especially in women. Evidence from Finland in 1996–2021
Source: Ann Med. 2024 Nov 26;56(1):2424455. doi: 10.1080/07853890.2024.2424455 (PMC11610349; doi:10.1080/07853890.2024.2424455)
Supplement: Supplemental Material [file IANN_A_2424455_SM1708.docx]

**Supplemental Material**

**eTable1: Terms and abbreviations used in the manuscript and their definitions.**

| **Term** | **Abbreviation** | **Definition** |
| --- | --- | --- |
| Acute myocardial  ischaemic syndrome | AMIS | Includes the first non-fatal and fatal myocardial ischaemic events.   Non-fatal myocardial ischaemic events include hospitalizations for MI or unstable angina. ICD codes: ICD-9: 410; ICD-10: I20.0, I21-I22.  Fatal myocardial ischaemic events includes death with coronary artery disease, cardiac arrest, sudden death for unknown reason, or unwitnessed death as the underlying or direct cause of death, or deaths with MI as the contributing cause of death.  ICD codes: ICD-9: 410-414, 798, not 7980A; ICD-10: I20-I25, I46, R96, R98. |
| Incidence (of AMIS) | - | The first myocardial ischaemic events (non-fatal and fatal, including out-of-hospital deaths) in Finnish population expressed as age-standardized event rate per 100,000 inhabitants per year.  The first event is defined as not having prior events in the last 10 years based on the national hospital discharge register. ICD codes listed above in the AMIS -section. |
| Mortality (of AMIS) | - | The fatal myocardial ischaemic events in Finnish population including out-of-hospital deaths expressed as age-standardized mortality rate per 100,000 inhabitants per year. The event was defined as fatal if the patient died within 28-days after the onset of the event. The ICD-codes for fatal events are listed above in the AMIS-section. The event was defined as the first if the patient did not have prior events in the last 10 years based on the national hospital discharge register. |
| Case fatality (of AMIS) | CF | The proportion (%) of all myocardial ischaemic events that resulted in death within 28 days after the onset of the event. Expressed as age-standardized to the age distribution of MI and stroke patients in the WHO MONICA Project. Only the first events are included in the present manuscript (i.e., no prior events in the last 10 years). ICD codes are listed above in the AMIS -section. |

**eTable 2:** The weights for age-standardizing acute myocardial ischaemic syndrome case fatality. Based on the age distribution of observed c*oronary events and stroke in the MONICA populations in the WHO-MONICA project and for the older age groups weights derived previously at the Finnish Institute for Health and Welfare^1^.*

| **Age (years)** | **Weights** |
| --- | --- |
| 0-4 | 1 |
| 5-9 | 1 |
| 10-14 | 1 |
| 15-19 | 1 |
| 20-24 | 1 |
| 25-29 | 1 |
| 30-34 | 2 |
| 35-39 | 5 |
| 40-44 | 9 |
| 45-49 | 16 |
| 50-54 | 26 |
| 55-59 | 42 |
| 60-64 | 56 |
| 65-69 | 75 |
| 70-74 | 93 |
| 75-79 | 100 |
| 80-84 | 100 |
| 85- | 100 |

**eTable 3:** The segmented (the Poisson or the negative binomial) regression model’s breakpoints and intervals of acute myocardial ischaemic syndrome incidence, mortality, and case fatality trends from 1996 to 2021. Intervals where there was no trend change are shown on a yellow background and increasing trends are shown on a red background.

| **Incidence** | | | | |
| --- | --- | --- | --- | --- |
| **Age group** | **Breakpoints** | **Interval 1 (estimate + slope [95% CI])** | **Interval 2 (estimate + slope [95% CI])** | **Interval 3 (estimate + slope [95% CI])** |
| **Men** |  |  |  |  |
| All (35-74) | 2003, 2009 | -10.398 - 0.009 [-0.020; 0.002] * year | -10.175 - 0.039 [-0.062; -0.017] * year | -10.521 - 0.013 [-0.018; -0.007] * year |
| 65-74 | 2002, 2012 | -8.383 - 0.018 [-0.029; -0.007] * year | -8.215 - 0.045 [-0.053; -0.038] * year | -8.636 - 0.018 [-0.025; -0.012] * year |
| 55-64 | 2004, 2007 | -8.880 - 0.018 [-0.027; -0.009] * year | -8.662 - 0.047 [-0.073; -0.020] * year | -9.045 - 0.013 [-0.017; -0.009] * year |
| 45-54 | 2003, 2009 | -10.924 - 0.006 [-0.004; 0.017] * year | -10.626 - 0.037 [-0.049, -0.027] * year | -11.052 - 0.005 [-0.010; 0.000] * year |
| 35-44 | 2001 | -13.973 + 0.010 [-0.015; 0.034] * year | -13.840 - 0.017 [-0.021; -0.012] * year | - |
| **Women** |  |  |  |  |
| All (35-74) | 2001, 2011 | -12.619 + 0.012 [-0.004; 0.028] * year | -12.367 - 0.035 [-0.043; -0.026] * year | -12.810 - 0.004 [-0.011; 0.003] * year |
| 65-74 | 2001, 2011 | -11.582 - 0.008 [-0.022; 0.006] * year | -11.360 - 0.051 [-0.059; -0.043] * year | -11.888 - 0.015 [-0.021; -0.011] * year |
| 55-64 | 2004, 2008 | -12.083 + 0.001 [-0.010; 0.011] * year | -11.567 - 0.064 [-0.120; -0.008] * year | -12.315 - 0.001 [-0.007; 0.004] * year |
| 45-54 | 2001, 2016 | -13.431 + 0.060 [0.024; 0.097] * year | -13.121 - 0.009 [-0.015; -0.002] * year | -13.881 + 0.029 [-0.007; 0.066] * year |
| 35-44 | 2000 | -16.447 + 0.069 [-0.009; 0.147] * year | -16.132 - 0.003 [-0.011; 0.006] * year | - |
|  |  |  |  |  |
| **Mortality** | | | | |
| **Men** |  |  |  |  |
| All (35-74) | 2020 | -12.377 - 0.046 [-0.048; -0.044] * year | -13.618 + 0.007 [-0.101; 0.114] * year | - |
| 65-74 | 2005, 2020 | -10.349 - 0.060 [-0.068; -0.051] * year | -10.504 - 0.042 [-0.046; -0.037] * year | -12.784 + 0.054 [-0.050; 0.016] * year |
| 55-64 | 2001, 2010 | -11.605 - 0.055 [-0.075; -0.036] * year | -11.720 - 0.032 [-0.043; -0.021] * year | -11.477 - 0.049 [-0.058; -0.040] * year |
| 45-54 | 2005, 2014 | -13.462 - 0.033 [-0.046; -0.020] * year | -13.170 - 0.067 [-0.083; -0.050] * year | -13.895 - 0.027 [-0.052; -0.001] * year |
| 35-44 | - | - | - | - |
| **Women** |  |  |  |  |
| All (35-74) | 2006 | -15.091 - 0.061 [-0.069; -0.053] * year | -15.258 - 0.044 [-0.050; -0.038] * year | - |
| 65-74 | 2006 | -14.738 - 0.079 [-0.088; -0.069] * year | -15.106 - 0.042 [-0.049; -0.035] * year | - |
| 55-64 | - | - | - | - |
| 45-54 | 2003, 2020 | -14.752 - 0.001 [-0.049; 0.046] * year | -14.320 - 0.065 [-0.082; -0.048] * year | -26.779 + 0.456 [-0.136; 1.048] * year |
| 35-44 | 2011 | -15.841 - 0.025 [-0.055; 0.005] * year | -14.442 - 0.119 [-0.192; 0.045] * year | - |
|  |  |  |  |  |
| **Case fatality** | | | | |
| **Men** |  |  |  |  |
| All (35-74) | 2002, 2010 | -2.557 - 0.060 [-0.069, -0.052] * year | -2.908 - 0.006 [-0.013, 0.002] * year | -2.473 - 0.036 [-0.041, -0.031] * year |
| 65-74 | 2004, 2010 | -2.059 - 0.061 [-0.070, -0.053] * year | -2.746 + 0.020 [0.002, 0.038] * year | -2.088 - 0.027 [-0.034, -0.020] * year |
| 55-64 | 2001, 2010 | -2.917 - 0.062 [-0.083, -0.042] * year | -3.228 - 0.000 [-0.013, 0.013] * year | -2.561 - 0.049 [-0.057, -0.041] * year |
| 35-54 | - | - | - | - |
| **Women** |  |  |  |  |
| All (35-74) | 2004, 2009 | -2.881 - 0.075 [-0.088, -0.063] * year | -3.491 + 0.004 [-0.017, 0.026] * year | -2.959 - 0.036 [-0.045, -0.028] * year |
| 65-74 | 2004, 2010 | -3.380 - 0.085 [-0.099, -0.070] * year | -4.142 + 0.012 [-0.016, 0.04] * year | -3.582 - 0.029 [-0.040, -0.019] * year |
| 55-64 | 2012 | -3.724 - 0.021 [-0.030, -0.012] * year | -3.244 - 0.050 [-0.077, -0.023] * year | - |
| 35-54 | - | - | - | - |

**eTable 4:** Average annual changes in acute myocardial ischaemic syndrome non-fatal incident incidence from 1996 to 2021. Incidence rate trends were analyzed using the Poisson or the negative binomial regression model. The rate changes are presented as average annual change percentages during the study period. P-values represent the significance of the trend by study year.

| **Incidence** | | | | |
| --- | --- | --- | --- | --- |
| **Age group** | **Average annual change (%)** | **95% CI** |  | **P-value** |
| **Men** | | | | |
| All (35-74)* | -1.16 | -1.37 to -0.95 |  | 5.70e-27 |
| 65-74* | -2.35 | -2.55 to -2.16 |  | 3.56e-123 |
| 55-64* | -1.25 | -1.44 to -1.06 |  | 1.09e-35 |
| 45-54* | -0.47 | -0.67 to -0.26 |  | 6.26e-6 |
| 35-44 | -0.44 | -0.77 to -0.12 |  | 0.008 |
| **Women** |  |  |  |  |
| All (35-74)* | -0.92 | -1.11 to -0.71 |  | 1.16e-18 |
| 65-74* | -2.48 | -2.69 to -2.27 |  | 6.27e-117 |
| 55-64* | -0.97 | -1.24 to -0.71 |  | 1.36e-12 |
| 45-54 | 0.96 | 0.64 to 1.29 |  | 5.65e-9 |
| 35-44 | 1.57 | 0.86 to 2.29 |  | 1.35e-5 |

*The negative binomial regression model was used instead of the Poisson regression model.

**eTable 5:** Sex, year, age and age-sex interaction variables and their effects on the incidence, mortality and case fatality trends of acute myocardial ischaemic syndrome. Incidence and mortality were analyzed using the negative binomial model. Case fatality was analyzed using the logistic regression model.

| **Incidence** | | | | | |
| --- | --- | --- | --- | --- | --- |
| **Age group** | **Parameter** | **Estimate** | **Standard error** | **z value** | **p-value** |
| 35–74 | Sex (male) | 1.2105 | 0.0011 | 108.43 | < 1e-16 |
|  | Year | -0.0184 | 0.0007 | -26.81 | < 1e-16 |
|  | Age | 0.1113 | 0.0008 | 142.40 | < 1e-16 |
|  | Age * Sex (male) | -0.0184 | 0.0010 | -18.56 | < 1e-16 |
|  |  |  |  |  |  |
| **Mortality** | | | | | |
| 35–74 | Sex (male) | 1.6545 | 0.0178 | 92.72 | < 1e-16 |
|  | Year | -0.0468 | 0.0008 | -61.28 | < 1e-16 |
|  | Age | 0.1318 | 0.0012 | 105.89 | < 1e-16 |
|  | Age * Sex (male) | -0.0210 | 0.0014 | -14.76 | < 1e-16 |
|  |  |  |  |  |  |
| **Case fatality** | | | | | |
| 35–74 | Sex (male) | 0.5627 | 0.0181 | 31.029 | < 1e-16 |
|  | Year | -0.0290 | 0.0006 | -48.763 | < 1e-16 |
|  | Age | 0.0331 | 0.0012 | 26.978 | < 1e-16 |
|  | Age * Sex (male) | 0.0018 | 0.0014 | 1.303 | 0.193 |


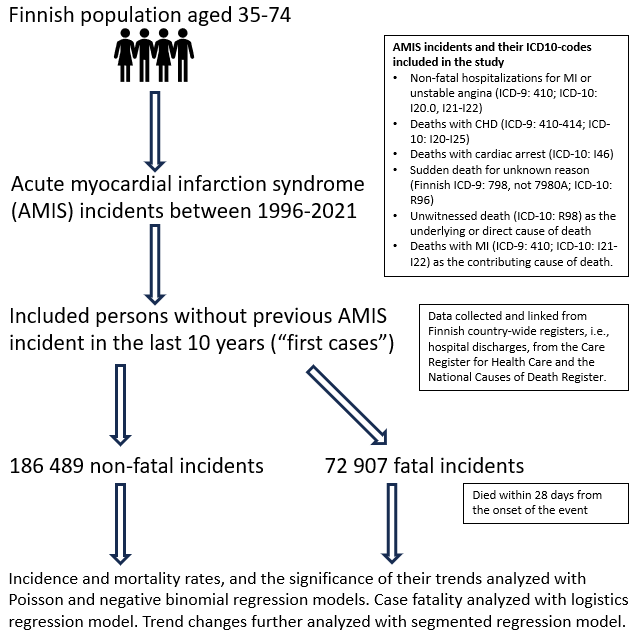


**eFigure 1: Flowchart of the study population and the methods.**

**
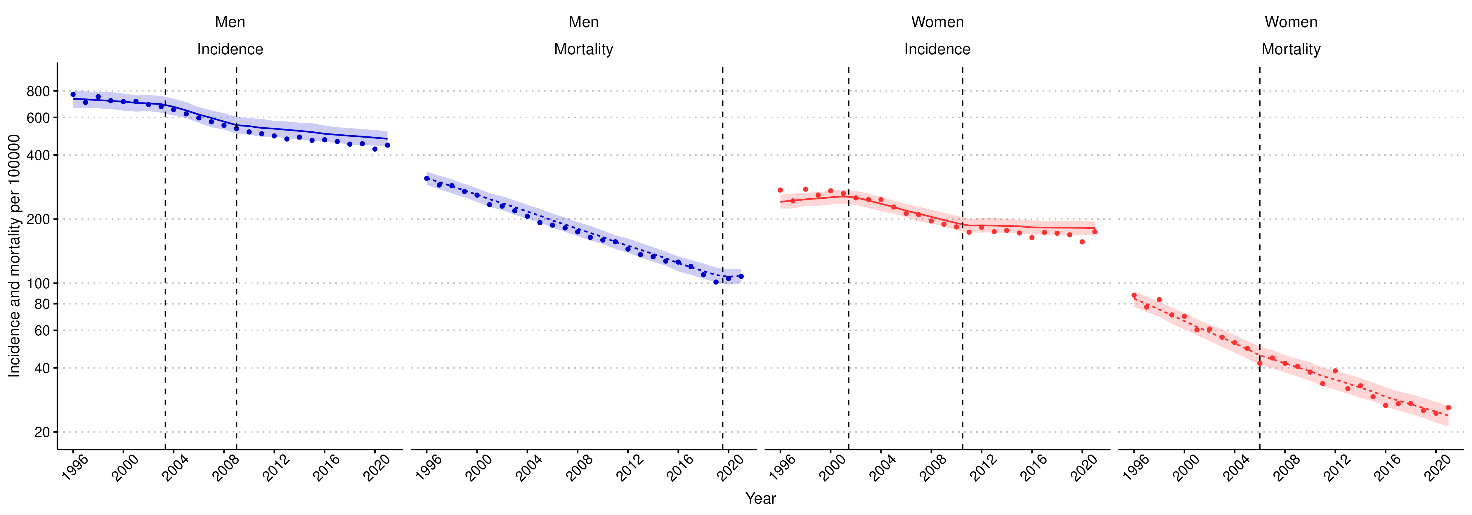
**

**eFigure 2: Trends in incidence and mortality rates of** acute myocardial ischaemic syndrome **in men and women aged 35-75 years, 1996-2021**. Rates include the first non-fatal I20.0, I21, and I22 cases and fatal I20-25, I46, R96 and, R98 cases. Age-standardized rates per 100 000 inhabitants were calculated with the 2011-2030 European standard population as the reference. Observed incidence and mortality rates are presented as dots, the segmented (negative binomial) regression model’s predicted values as a line, and the regression model’s 95%-confidence intervals for predictions as ribbons. Breakpoints with a significant trend change are marked as vertical dashed lines. The rates are presented on a logarithmic scale.

**
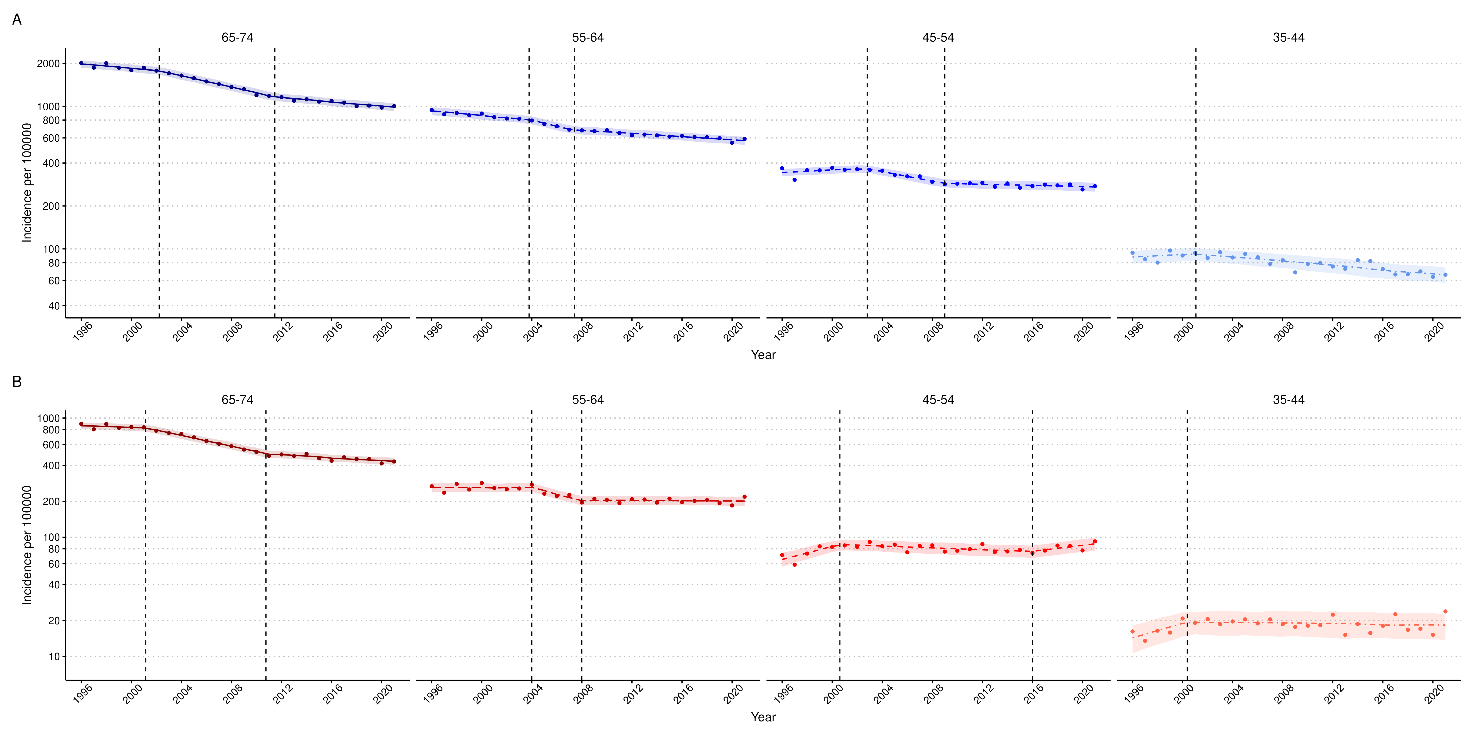
**

**
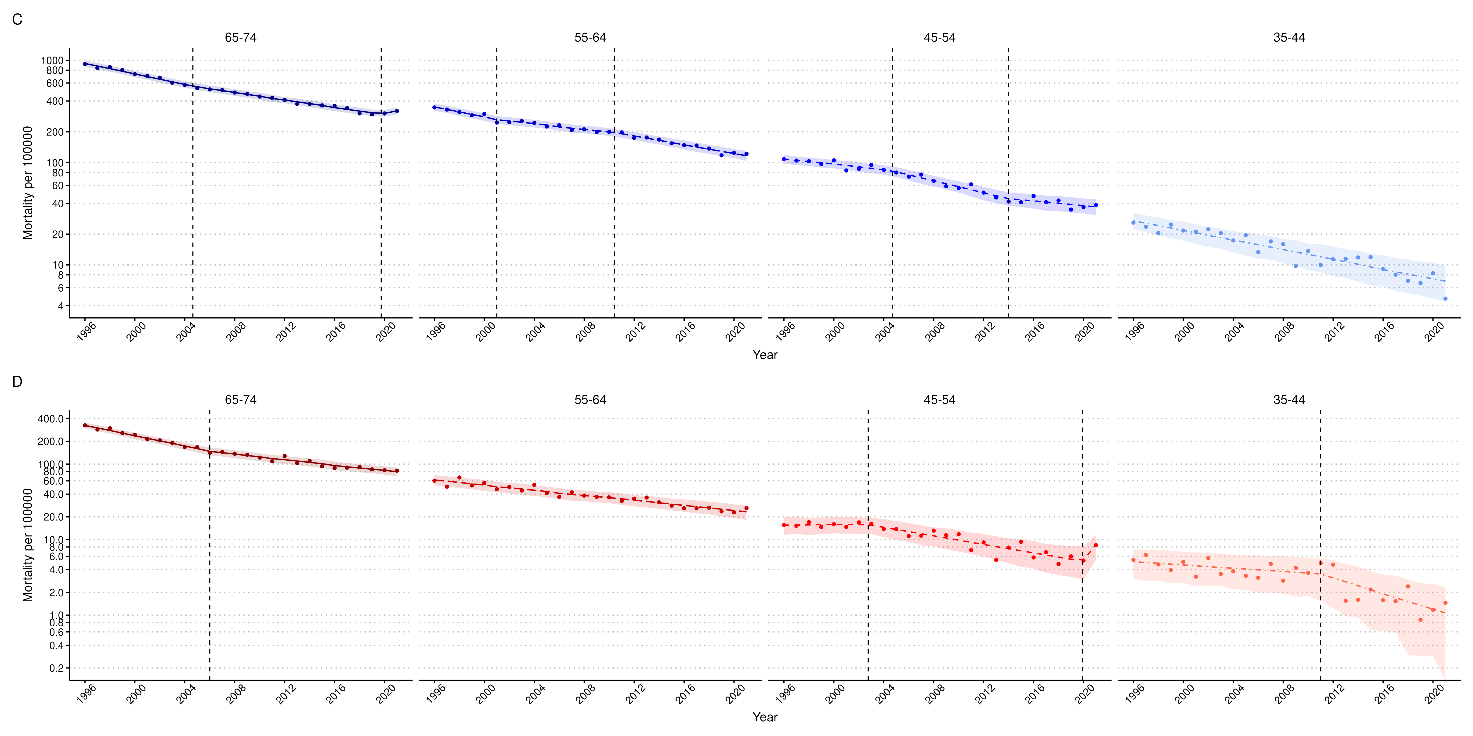
**

**eFigure 3: Trends in incidence and mortality rates of** acute myocardial ischaemic syndrome **in men and women by age group, 1996-2021.** A) Incidence rates of men, and B) women. C) Mortality rates of men, and D) women. The incidence rates include the first non-fatal I20.0, I21, and I22 and fatal I20-25, I46, R96, and R98 cases. Mortality rates include the first fatal cases as mentioned above. Age-standardized rates per 100 000 inhabitants were calculated with the 2011-2030 European standard population as the reference. The observed incidence and mortality rates are presented as dots, the segmented, the Poisson or the negative binomial regression model’s predicted values as a line, and the regression model’s 95%-confidence intervals for predictions as ribbons. Breakpoints with a significant trend change are marked as vertical dashed lines. The rates are presented on a logarithmic scale.


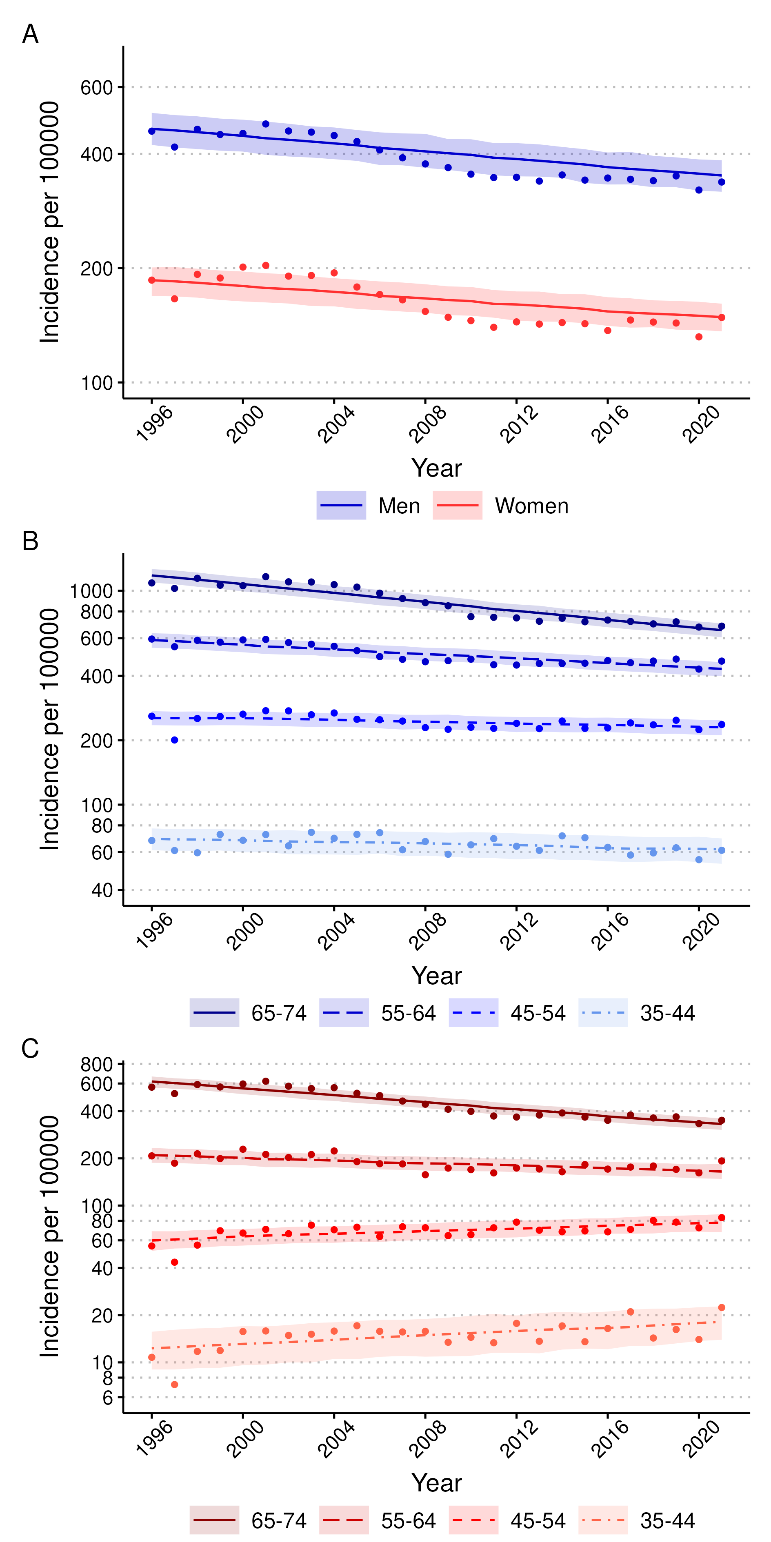


**eFigure 4: Trends in incidence of the first non-fatal acute myocardial ischaemic syndrome incidents in men and women by age group, 1996-2021.** A) Incidence rates of men and women aged 35-74. B) Incidence rates of men in 10-year age groups. C) Incidence rates of women in 10-year age groups. The incidence rates include first non-fatal I20.0, I21, and I22 cases. Age-standardized incidence rates per 100 000 inhabitants were calculated with the 2011-2030 European standard population as the reference. Observed incidences are presented as dots, the Poisson or negative binomial regression model’s predicted values as a line and the regression model’s 95%-confidence intervals for predictions as a ribbon. The rates are presented on a logarithmic scale.

**
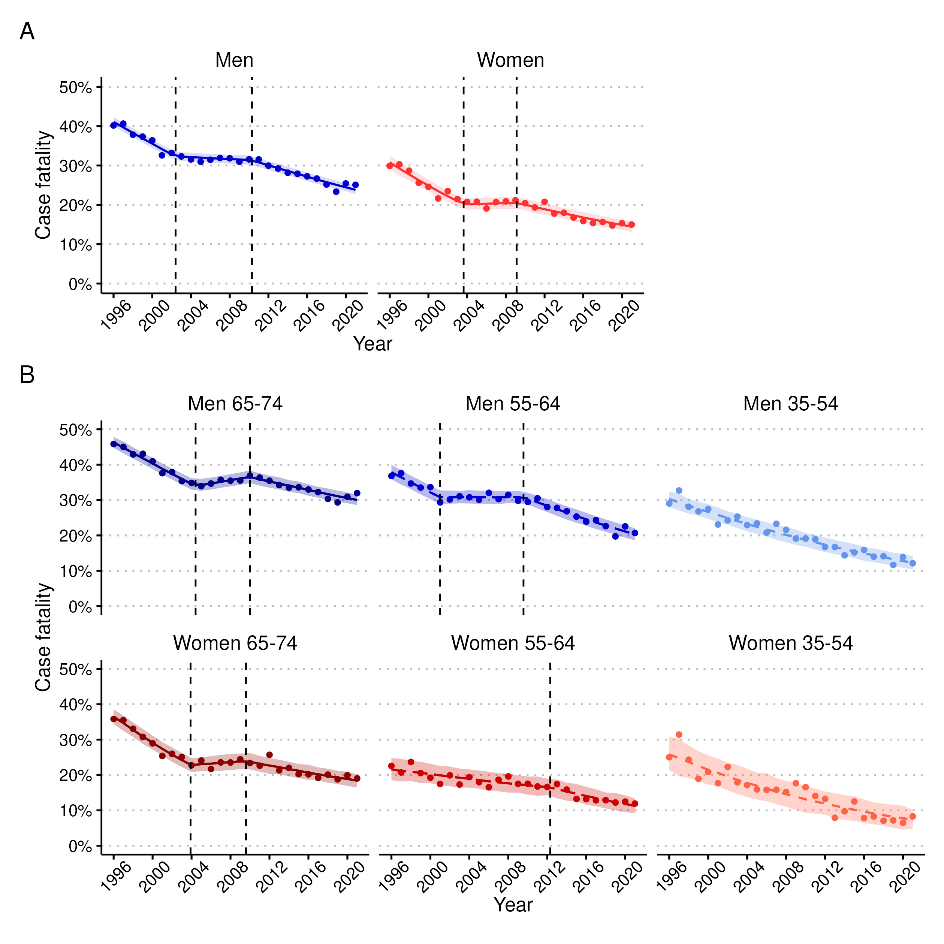
**

**eFigure 5: Trends in case fatality of acute myocardial ischaemic syndromeA) among men and women aged 35-74, and B) by 10-year age groups, 1996-2021.** The case fatality includes the first non-fatal I20.0, I21, and I22 and fatal I20-25, I46, R96, and R98 cases. The case fatality was age-standardized using weights based on the age distribution of observed myocardial ischaemic events in populations participating in the WHO-MONICA project. Observed case-fatality is presented as dots, the segmented or the logistic regression model’s predicted values as a line, and the regression model’s 95%-confidence intervals for predictions as ribbons. Breakpoints with a significant trend change are marked as vertical dashed lines.

**References:**

1. Koukkunen H, Havulinna AS, Lehto S, et al. Case fatality of acute coronary events is improving even among elderly patients; the FINAMI study 1995-2012. *Ann Med* 2018;50(1):35-45. doi: 10.1080/07853890.2017.1382713 [published Online First: 20171003]
